# Diurnal rhythms of urine volume and electrolyte excretion in healthy young men under differing intensities of daytime light exposure

**DOI:** 10.1038/s41598-021-92595-0

**Published:** 2021-06-23

**Authors:** Isuzu Nakamoto, Sayaka Uiji, Rin Okata, Hisayoshi Endo, Sena Tohyama, Rina Nitta, Saya Hashimoto, Yoshiko Matsushima, Junko Wakimoto, Seiji Hashimoto, Yukiko Nishiyama, Dominika Kanikowska, Hiromitsu Negoro, Tomoko Wakamura

**Affiliations:** 1 Human Health Sciences, Graduate School of Medicine, Kyoto University, 53 kawahara-cho, shogoin, sakyo-ku, Kyoto, 606-8507 Japan; 2 Human Health Sciences, Faculty of Medicine, Kyoto University, Kyoto, Japan; 3 Clinical Laboratory, Kyoto University Hospital, Kyoto, Japan; 4 Department of Pathophysiology, Poznan University of Medical Sciences, Poznan, Poland; 5 Department of Urology, University of Tsukuba, Ibaraki, Japan

**Keywords:** Physiology, Metabolism

## Abstract

In humans, most renal functions, including urine volume and electrolyte excretions, have a circadian rhythm. Light is a strong circadian entrainment factor and daytime-light exposure is known to affect the circadian rhythm of rectal temperature (RT). The effects of daytime-light exposure on the diurnal rhythm of urinary excretion have yet to be clarified. The aim of this study was to clarify whether and how daytime exposure to bright-light affects urinary excretions. Twenty-one healthy men (21–27 years old) participated in a 4-day study involving daytime (08:00–18:00 h) exposure to two light conditions, Dim (< 50 lx) and Bright (~ 2500 lx), in a random order. During the experiment, RT was measured continuously. Urine samples were collected every 3 ~ 4 h. Compared to the Dim condition, under the Bright condition, the RT nadir time was 45 min earlier (*p* = 0.017) and sodium (Na), chloride (Cl), and uric acid (UA) excretion and urine volumes were greater (all *p* < 0.001), from 11:00 h to 13:00 h without a difference in total daily urine volume. The present results suggest that daytime bright light exposure can induce a phase shift advance in urine volume and urinary Na, Cl, and UA excretion rhythms.

## Introduction

Most organisms have circadian systems that are associated with the earth’s 24-h light–dark cycle and adapted to their particular niches. In humans, renal functions including renal blood flow, glomerular filtration rate (GFR), urine volume, and some electrolyte excretion are highest from afternoon to early evening and lowest at midnight^1^. These circadian rhythms persist independent of meal timing, activity level, sleep, and body posture, indicating that they are driven by an internal time-keeping system^2–5^. In most entrained 24-h studies, subject lives in a laboratory with constant meal time, timed activities, and sleep deprivation (i.e., the constant routine). However, it is necessary to examine how circadian rhythms are affected with sleep–wake rhythm in real life that is not such a restricted life.

The primary role of the kidney is to support homeostasis by eliminating waste products through glomerular filtration, reabsorption, and excretion of water and electrolytes in the renal tubules. Kidney function is closely associated with blood pressure (BP) regulation, and the renin–angiotensin–aldosterone system modulates daily BP variation through physiological mechanisms that regulate water and sodium (Na)^6^. As well known, Arginine vasopressin, the antidiuretic hormone, regulates the circadian rhythm of urine volume^7^.

In mammals, the suprachiasmatic nucleus (SCN) of the hypothalamus serves as a master circadian clock. This body clock is constituted by oscillation-generating neurons. The resultant circadian rhythms are fundamentally genetic, but also regulated by external entrainment factors, such as the light–dark cycle, eating, and exercise.

The light–dark cycle is the most profound synchronizer of SCN activity^8–11^, with the magnitude and direction of circadian resetting responses being dependent on the circadian phase during which light stimulation is present^12–15^. Rectal temperature (RT) and melatonin rhythms are peripheral indicators of the timing of the human circadian pacemaker^8,14^. Bright daytime light (6000 lx) exposure from wake-up time to sunset can induce an earlier RT nadir time in humans^16^. Furthermore, compared to dim daytime light exposure, daytime light (5000 lx) exposure has been reported to result in greater nocturnal melatonin secretion^17^.

Given that bright light exposure during daytime hours influences diurnal rhythms for RT and melatonin levels, we suspect that it may also affect the diurnal rhythm of urinary excretion. Additionally, there is a need to examine how diurnal rhythms are altered in a real life-like context given that in most 24-h entrainment studies, subjects live in a laboratory-controlled environment with a consistent meal-time and activity routine as well as a sleep deprivation component. The aim of the present study was to compare how bright versus dim light exposure during the daytime affects the diurnal rhythms of urine volume, electrolyte levels, and uric acid (UA) excretion in human subjects.

## Materials and methods

### Participants

This study was conducted in accordance with the guidelines established in the Declaration of Helsinki, approved by the Ethics Committee of Kyoto University Graduate School, and registered in the University Hospital Medical Information Network database (UMIN000038350). Twenty-one healthy young men (21–27 years old) were recruited from the authors’ university between November 2019 and February 2020. Prior to participating in this study, participants received a detailed explanation of the purpose and design of the study and then provided written informed consent.

Exclusion criteria were sleep disorders as determined by the Pittsburgh Sleep Quality Index^18^ or being extreme morning or evening types as determined by Morningness–Eveningness Questionnaire^19^. According to their Cornell Medical Index values^20^ and International Prostate Symptom Scores^21^, all participants were healthy and had no urination problems, respectively. None of the participants were tobacco smokers, taking medications, had food allergies, or had traveled across time zones within 3 months prior to the start of the experiment.

### Study protocol

**Figure 1:**
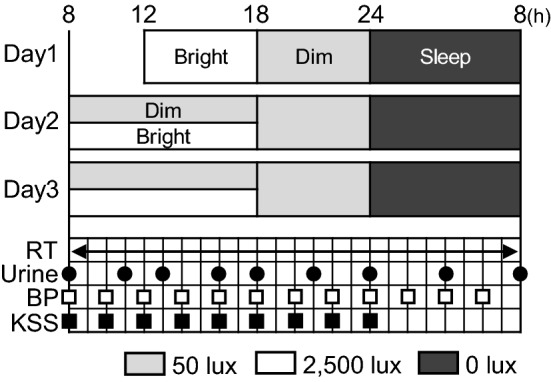
Overview of the experimental protocol. Numbers above the graphic indicate hours in the day that served as measurement period boundaries. RT was monitored continuously (arrow). Black circles indicate approximate urine sample collection times. White squares indicate BP measurement time points. Black squares indicate KSS implementations.

An overview of the experimental protocol is shown in Fig. 1. For the 5 days preceding the experiments, participants were asked to go to bed at 24:00 ± 1 h and to get up at 07:00 ± 1 h. Their adherence to this schedule was checked via sleep diaries.

The experiments were conducted in a temperature (25 ± 2 °C) and humidity (55 ± 5%) controlled experimental room with a bed, table, and full restroom. Participants spent two periods (4 days and 3 nights each) in the experimental room under different daytime light conditions (dim or bright). The order of the periods was randomized across subjects with at least 5 days between the dim/bright and bright/dim experimental periods. The daytime hours during which these conditions were applied spanned from 08:00 h to 18:00 h.

The dim condition was < 50 lx (21.94 μW/cm^2^) and the bright condition was ~ 2500 lx (769.77 μW/cm^2^). Light intensity was measured vertically at eye level by a seated researcher with a Lighting Passport spectrometer (Asensetek Inc., Taipei, Taiwan). The light sources were fluorescent lamps that illuminated the experimental room via a pseudo-window. Estimated irradiances were calculated with the Irradiance Toolbox^22^.

On the first experimental day, participants were brought to the experimental room at 12:00 h and then allowed to acclimate to the room environment. In the evening of day 1, they were kept in dim light from 18:00 h to 24:00 h, and then left in darkness from 24:00 h to 08:00 h to sleep. On subsequent days, they were exposed to dim or bright light from 08:00 h to 18:00 h and to dim light from 18:00 h to 24:00 h. At 08:00 h on day 2, the participants were asked to urinate to ensure that each started the experiment with an empty bladder. Subsequently, they were asked to collect their urine in a P-Flowdiary portable uroflowmetry device (Micronix Inc., Kyoto, Japan) to measure urine volume. Participants were asked to inform an experimenter when they needed to urinate and to place collected urine samples at the entrance to the experimental room. On day 3, participants were asked to collect urine every 3 ~ 4 h.

Identical meals (each 700 kcal, 60% from carbohydrates, 25% from fat, and 15% from protein; 3.8 g salt) were provided to participants at 08:00 h, 13:00 h, and 18:00 h. Participants consumed 25 ml/kg body weight per day of water every 2 h from 08:00 h to 24:00 h. Overall, the mean volume of water consumed each day was 1621 ± 171 ml. Participants were allowed to read books, to listen to music, and to use portable devices (smartphones and laptops) with a screen illuminance level < 50 lx. Although they were not expected to follow a strict daily routine, to prevent extremes in activity, participants were not permitted to engage in strenuous physical exercise or to take a nap. During the experiment, the participants wore a short-sleeved T-shirt and shorts without socks. Each took a shower for up to 30 min between 15:00 and 16:00 h.

### Measurements

RT was measured continuously and reported once per minute with an NT data logger (N543R, Nikkiso-Therm Co., Tokyo, Japan). BP and heart rate (HR) were measured every 2 h with a HEM-7080IC monitor (Omron Healthcare Co., Kyoto, Japan). The RT, BP, and HR data were extracted with a computer. Participants filled out a Kwansei Gakuin Sleepiness Scale (KSS) of subjective sleepiness every 2 h from 08:00 h to 24:00 h. A higher KSS score indicates being sleepier (range 0–7).

Urinalysis was conducted to determine urine osmolality and to measure Na, K, chloride (Cl), calcium (Ca), phosphorus (P), magnesium (Mg), and UA concentrations. Urine samples collected were each transferred from a P-Flowdiary portable to a Urinemate P partition cup (Sumitomo Bakelite Co., Ltd., Tokyo, Japan), which enables 1/50th of a urine volume to be collected accurately and proportionally. Promptly after being collected, a 10-ml portion of each urine sample was centrifuged at 3000 rpm for 5 min; the supernatant was transferred into plain tubes and stored at 4 °C until being submitted to urinalysis by the Kyoto University Hospital clinical laboratory with a TBA-2000FR automatic biochemical analyzer (Canon Medical Systems Corporation, Tochigi, Japan).

### Statistical analysis

Because two of the participants were excluded due missing data values, 19 participants were analyzed. Unless otherwise specified, data are reported as means ± standard errors of the mean (SEMs). To obtain diurnal rhythm nadir times and amplitudes, Cosinor analysis^23^ of RT data was conducted in Time Series Analysis Serial Cosinor 8.0 software (Expert Soft Technologies, France). Differences in RT nadir time and amplitude and in 24-h urine excretion values between the two daytime light conditions were analyzed with paired *t*-tests. Data from 3 participants were excluded from the 24-h urinalysis because of collection errors with Urinemate P partition cup operation, leaving a final *N* of 16 for the 24-h urinalysis variables. Urinary excretion values obtained from samples collected every 3–4 h were converted to volumes per hour. Then urine volume was categorized according to the following bins: daytime, 08:00–18:00 h; evening, 18:00–24:00 h; and sleep, 24:00–08:00 h. Linear mixed modeling was performed with light condition and time as fixed factors and with subject as a random factor. When significant interaction effects were detected, Bonferroni post hoc tests were applied. BP and HR values were converted to in % of the individual 24-h means. Because seven of the participants were excluded due missing night time BP data, 12 participants were analyzed. A two-way repeated-measures ANOVA was performed. When significant interaction effects were detected, Bonferroni post hoc tests were applied. Pearson’s correlation analysis was used to examine whether RT nadir time difference and urine volume difference correlations were affected by light condition. Data analyses were performed in SPSS version 24 for Windows (IBM Japan Inc., Tokyo, Japan) with a significance criterion of *p* < 0.05.

## Results

### Participant characteristics

The characteristics of the recruited cohort (*N* = 19), reported as means ± standard deviations (SDs) were: age, 23.7 ± 1.7 years; body weight, 64.8 ± 6.3 kg; height 174.2 ± 5.7 cm; body mass index 21.4 ± 1.5 kg/m^2^; Pittsburgh Sleep Quality Index scores, 3.8 ± 1.9 points; and Morningness-Eveningness Questionnaire scores, 50.8 ± 8.9 points.

### Effects of illumination brightness on RT and urine excretion

**Figure 2:**
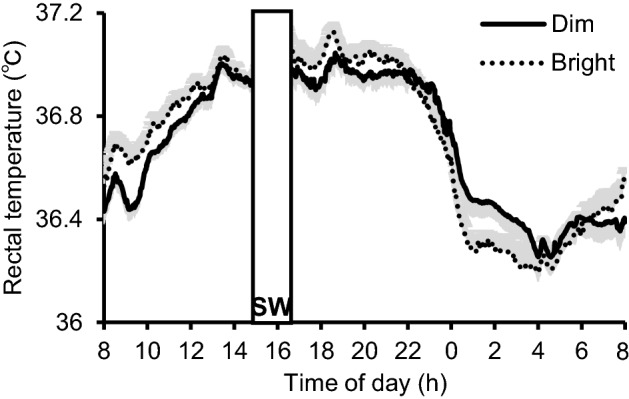
Mean diurnal rhythms in RT under the two light conditions. SW, no data due to shower time. Light grey shading indicates SEM. *N* = 19.

RT nadir times, RT amplitudes, urine volumes, and urine constituent concentrations are compared between the bright and dim daytime conditions in Table 1. At baseline on day 1, there was no difference in mean RT nadir time and mean RT amplitude in two light conditions. Notably, mean RT nadir time was significantly earlier (by 45 min) and mean RT amplitude was significantly greater (by 22%) in the bright condition than in the dim condition. Mean RT values are shown in Fig. 2. Mean 24-h urinary excretion values did not differ between the two conditions for urine volume or for Na, K, Cl, Ca, P, Mg, or UA levels (Table 1).

**Table 1:** Comparison of rectal temperature (RT) profile (*N* = 19) and 24-h urinary excretion variables (*N* = 16) between light condition groups.

Variable	Condition	Mean ± SEM	t	*p*
RT nadir time, h On the day 1	Dim	5:31 ± 0.54	0.39	0.701
	Bright	5:22 ± 0.51		
RT amplitude, °C On the day 1	Dim	0.38 ± 0.01	0.35	0.729
	Bright	0.37 ± 0.01		
RT nadir time, h On the day 3	Dim	5:37 ± 0.30	2.63	0.017
	Bright	4:52 ± 0.28		
RT amplitude, °C On the day 3	Dim	0.37 ± 0.03	2.43	0.026
	Bright	0.45 ± 0.04		
Urine volume, ml/day	Dim	1657 ± 75.3	0.81	0.433
	Bright	1711 ± 72.8		
Na, mEq/day	Dim	146 ± 5.3	1.14	0.272
	Bright	155 ± 6.3		
K, mEq/day	Dim	24.8 ± 2.0	0.77	0.454
	Bright	23.3 ± 1.2		
Cl, mEq/day	Dim	125 ± 4.5	0.78	0.446
	Bright	131 ± 5.4		
Ca, mg/day	Dim	172 ± 13.9	0.04	0.965
	Bright	172 ± 14.3		
P, mg/day	Dim	690 ± 33.7	0.61	0.548
	Bright	680 ± 26.2		
Mg, mg/day	Dim	82.8 ± 4.3	0.21	0.837
	Bright	83.2 ± 4.6		
UA, mg/day	Dim	440 ± 12.2	1.21	0.247
	Bright	453 ± 16.5		

*Ca* calcium, *Cl* chloride, *K* potassium, *Mg* magnesium, *Na* sodium, *P* phosphorus, *RT* rectal temperature, *UA* uric acid. Statistical values are from paired *t*-tests.

**Figure 3:**
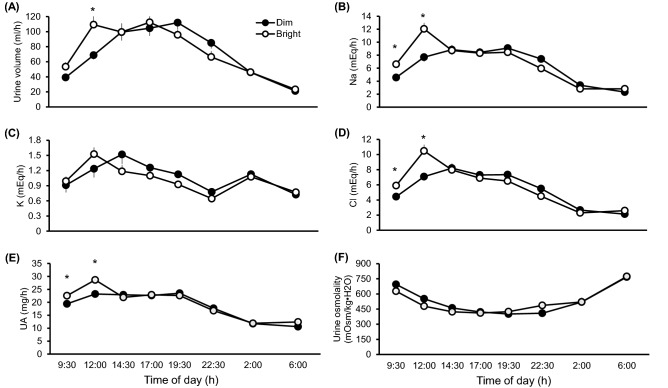
Effects of bright versus dim daytime light exposure on urine excretion variables. (**A–F**) Mean urine volume, Na, K, Cl, UA, and urine osmolality values ± SEMs, respectively, over time. Linear mixed model and Bonferroni post hoc tests were used (*N* = 19); *p < 0.05.

Analysis of changes in urine constituents across time-of-day intervals revealed a significant main effect of time of day on urine volume (F_7, 268_ = 31.50; *p* < 0.001), on Na, K, Cl, Ca, P, Mg, and UA concentrations (F_7, 268_ = 46.70; *p* < 0.001, F_7, 268_ = 13.39; *p* < 0.001, F_7, 268_ = 48.35; *p* < 0.001, F_7, 268_ = 75.14; *p* < 0.001, F_7, 268_ = 168.25; *p* < 0.001, F_7, 268_ = 64.41; *p* < 0.001, and F_7, 268_ = 58.88; *p* < 0.001, respectively), and on urine osmolality differences between the dim and bright condition (F_7, 268_ = 34.58; *p* < 0.001) (Fig. 3 and Supplementary Fig. 1). Significant interactions were observed for urine volume, Na, Cl, and UA (F_7, 268_ = 2.66; *p* = 0.011, F_7, 268_ = 5.37; *p* < 0.001, F_7, 268_ = 4.51; *p* < 0.001, F_7, 268_ = 2.72; *p* = 0.010, respectively). Post hoc tests revealed that, compared to the dim condition, the bright condition was associated with greater excretion of Na (*p* = 0.012), Cl (*p* = 0.035), and UA (*p* = 0.026) during the morning period (08:00–11:00 h) as well as higher excretion of urine volume, Na, Cl, and UA during midday period (11:00–13:00 h; all *p* < 0.001).

### Relationship between light exposure effects on RT nadir time and light exposure effects on urine volume and participants’ characteristics

**Figure 4:**
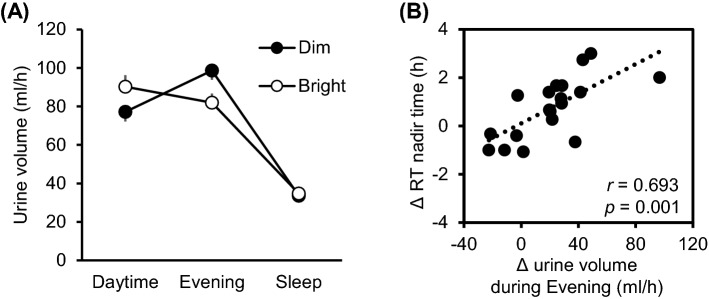
Effects of daytime light exposure condition on urine volume and analysis of the relationship between inter-condition differences in RT nadir time and inter-condition differences in evening-period urine volumes (both, *N* = 19). (**A**) Mean urine volumes collected (± SEMs) during daytime hours (08:00–18:00 h), evening hours (18:00–24:00 h), and sleeping hours (24:00–08:00 h). (**B**) Differences in RT nadir time between the dim and bright light conditions correlated very significantly with differences in evening-period urine volumes between the two light conditions. Differences were calculated by subtracting bright condition values from dim condition values within each noted time interval on day 3. Pearson correlation statistics are shown within the correlation graph.

As shown in Fig. 4, the differences in RT nadir time between the dim and bright light conditions correlated significantly with differences in urine volume between these two light conditions during the evening (*r* = 0.693, *p* = 0.001), but not during the daytime (*r* = 0.049, *p* = 0.841). The differences in RT nadir time between the dim and bright light conditions were not correlated with PSQI scores, MEQ scores, and BMI (*r* = − 0.023; *p* = 0.925, *r* = − 0.140; *p* = 0.567, *r* = − 0.026; *p* = 0.917, respectively).

### Cardiovascular variables and subjective sleepiness

**Figure 5:**
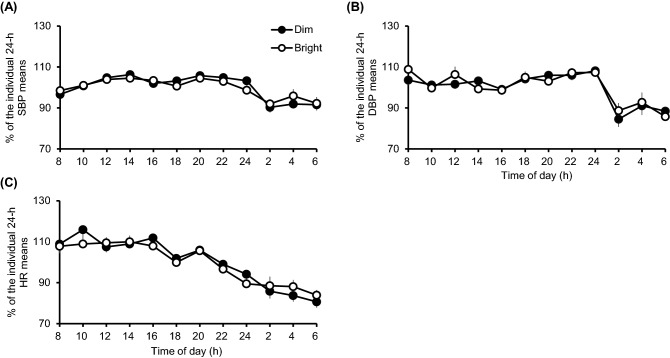
A two-way repeated-measures ANOVA analyses on % of the individual 24-h means in BP and HR. (**A–C**) % of Systolic BP (SBP), diastolic BP (DBP), and HR, respectively, were each very significantly affected by the time of day, but not by light condition. There were no significant time × light condition interactions; means ± SEMs are shown (*N* = 12).

As shown in Fig. 5 and Supplementary Fig. 2, significant main effects of time-of-day on % of the individual 24-h SBP means, % of the individual 24-h DBP means, % of the individual 24-h HR, and KSS score in dim and bright conditions were observed (F_11, 264_ = 10.02; *p* < 0.001, F_11, 264_ = 10.42; *p* < 0.001, F_11, 264_ = 25.09, F_8, 306_ = 23.13; *p* < 0.001, respectively). There were no significant main effects of light condition on these variables, nor were there any significant time × light interactions. Diurnal rhythm in SBP, DBP, and HR are shown in Supplementary Fig. 3.

## Discussion

In the present study, we found that the level of light exposure one experiences during daytime hours (08:00–18:00 h) affected urine volume and urinary Na, Cl, and UA excretions. Compared to subjects exposed to dim daytime light (< 50 lx), subjects exposed to bright daytime light (~ 2500 lx) had higher mid-day (11:00–13:00 h) excretion values, an earlier RT nadir, and an increased RT amplitude. Moreover, a more pronounced shift in RT nadir time was associated with a more pronounced difference in urine volume. These data are consistent with the expectation that level of light exposure during daytime hours can affect diurnal regulation of physiology. To the best of our knowledge, this is the first study that has investigated the effects of daytime light conditions on the diurnal rhythms of urinary excretions, and the relationship of such an effect with RT.

Under both light conditions, urinary excretions exhibited a clear diurnal rhythm, being high during wakefulness and low during sleep, with the exception of urinary P excretion, which increased from the evening period to the sleep period. Urinary osmolality was consistently low during daytime hours. These observations are consistent with those of several previous studies^4,24–26^. Notably, we found that daytime light exposure affected the diurnal rhythms of urine volume and of urinary Na, Cl, and UA concentrations. Moreover, the magnitude of the bright light-induced RT phase shift correlated with bright light-induced diminution of urine volume, especially during the evening (18:00–24:00). Notwithstanding, there were no differences in total 24-h excretion volumes or electrolyte levels between the two daytime light exposure conditions. Hence, the effects of daytime light exposure on the variables analyzed in the study did not affect homeostasis, but rather induced a phase advance in urine volume and urinary Na, Cl, and UA excretion rhythms.

The mechanisms mediating the diurnal rhythm of urine excretion have not been elucidated in detail, likely because the regulation urine excretion and electrolyte levels is complex. The aforementioned phase advance associated with bright daytime light exposure could reflect an increased GFR, which in turn would be expected to result in increased urine volume and increased Na and Cl excretions (Fig. 3). However, an increase in GFR cannot explain why urinary K excretion was not increased in the bright light condition given that K excretion has a diurnal rhythm similar to Na excretion^27^.

A change in GFR is related specifically to the first of three phases of urine production, namely glomerular filtration, and does not account for changes affecting the subsequent phases of reabsorption and secretion. Hence, it is possible that the differing effects of daytime light exposure on the excretion of particular electrolytes reflect distinct influences on particular stages. For example, a modulatory effect on secretion and/or reabsorption within the renal tubule would be independent of overall GFR per se. The main hormones involved in natriuresis (Na excretion) are natriuretic peptides, including atrial natriuretic peptide, and aldosterone. Given that plasma concentrations of atrial natriuretic peptide and aldosterone have been shown to have diurnal rhythms^28^, we suspect that these hormones may have been involved in mediating the presently observed bright light exposure-related shift in natriuresis.

Diurnal rhythms of urine excretions are induced, at least in part, from diurnal changes in secretion and reabsorption capacities of the distal nephron and collecting ducts. Recent studies on the role of circadian clock proteins in the regulation of genes involved in renal transport suggest that circadian fluctuations in renal function are controlled partly by a molecular clock in the kidney^11,29–31^. In the present study, daytime light exposure effects on the SCN may have affected the peripheral clock in the kidney as like the advancement of RT rhythm. Another possibility is that the acute effect of bright light may have enhanced urine excretions and volume in the morning not correlated with the circadian clock. However, such an effect has not been published as far as we know. This effect can be proven by an experiment of the dim light condition followed by the bright light exposure. It is valuable to examine the mechanism of how bright light exposure affects diurnal variation in urine excretions and volume in the future experiment.

Peripheral circadian rhythms can be influenced by the SCN via a combination of neural, hormonal, and body temperature signals^32,33^. Melatonin has been shown to facilitate aldosterone production in human adrenocortical cells^34^. Released aldosterone, which acts to increase renal tubular reabsorption of Na and water, has a circadian rhythm characterized by an increase through the night until it peaks in the morning^35,36^. Although melatonin and aldosterone were not measured in the present study, we would expect that morning melatonin levels would have been decreased under the present bright light condition because bright light exposure decreases melatonin secretion during morning hours, compared to dim light^16,37^. Hence, we posit that lower melatonin and aldosterone levels in subjects exposed to the bright condition, compared to those exposed to the dim condition, may elevate urinary Na excretion, especially in the morning (Fig. 3).

With respect to UA excretions, it is difficult to apply rodent UA excretion data to humans because, relative to rodents, humans have much lower expression of uricase, an enzyme that metabolizes UA to allantoin^38^. The relatively high serum UA levels found in humans has been suggested to serve an antioxidant role^39^. If so, the higher early-day (08:00–13:00 h) UA excretions observed in the bright condition than in the dim condition might reflect a physiological upregulation of UA for the purpose of activating its antioxidant effect. Melatonin has an antioxidant effect as well^40^. Therefore, daytime bright light exposure induced stimulation of melatonin secretion in the evening might affect antioxidant activity in addition to facilitating sleep. These are intriguing possibilities worthy of further research.

Though light sensitivity differed between individuals (Fig. 4), the individual response to different light intensities were not associated with PSQI, MEQ, or BMI. Individual traits such as age, sex, chronotype, race/ethnicity, genetic haplotypes have been reported for light sensitivity^41^, but the definitive ones are unknown. Evening-light exposure at even 10 lx beginning 4 h before bedtime results in later apparent melatonin onsets in the highest sensitivity participant^42^. However, the least sensitive participant did not achieve this level of suppression until exposed to the bright light of 400 lx. There are large interindividual differences in light sensitivity. Participants who did not advance the RT phase by daytime bright light exposure were considered to have a high sensitivity to evening light, and the dim evening light (50 lx) might delay the rectal RT phase. Notably, the difference in light sensitivity affected not only rectal RT but also a diurnal variation of urine volume.

Under both experimental light conditions, we observed normal diurnal rhythms for BP, HR, and subjective sleepiness (Fig. 5 and Supplementary Fig. 2). The subjects’ BP and HR values differed between waking hours (high) and nighttime hours (low), but did not differ between the two light conditions. All subjects, regardless of light exposure condition, reported low subjective sleepiness through the bulk of daytime hours. Interestingly, just 10 min of bright light exposure every 4 h has been shown previously to result in increased HR, relative to dim light exposure, from waking through the first 12 h after waking^43^. In addition, 20-min bright light exposure in the late morning (within the period of 10:00–12:00 h) enhanced muscle sympathetic nerve activity and increased HR^44^. In these previous studies, short-term light exposure activated sympathetic nerve activity and increased HR. However, there are no prior reports showing that continuous exposure to bright light for the bulk of one’s waking hours over multiple days (10 h/day for 3 days in this experiment) increases HR. Our not finding light exposure effects on HR suggest it did not affect sympathetic nerve activity or cardiovascular variables significantly and thus that short-term and long-term light exposure may have different effects on HR. Moreover, in prior studies that incorporated sleep deprivation, daytime bright light exposure (09:00–17:00 h, > 400 lx) increased systolic and diastolic BP at 16:00 h compared with values seen with dim light exposure (09:00–17:00 h, < 10 lx)^45^. Notably, the consistency of these variables across our experimental light exposure conditions suggests that the mental and physiological states of the participants were stable during this study. The fact that our subjects maintained a consistent sleep–wake cycle may have been a strong factor in sustaining their BP and HR values regardless of light exposure condition. Moreover, this consistency supports the reliability of the present results given that sleep deprivation leads to excessive natriuresis^32^.

The present study had several limitations. First, because this study was exploratory, blood electrolyte levels, hormonal levels (aldosterone, atrial natriuretic peptide, melatonin), and GFR were not measured. Therefore, our data cannot indicate which stages of urine production were influenced by light exposure and the detailed mechanisms mediating the presently reported effects remain to be resolved. Second, our results cannot be compared directly with data obtained with constant-routine protocols, which are generally used for internal circadian rhythm determination. We did not employ the constant-routine method because we aimed to evaluate diurnal rhythms under conditions that were closer to real-life conditions. Thirdly, a previous study has shown that habitual light exposure influence circadian rhythm phase and light sensitivity^46^, but habitual light exposure was not measured. Finally, because all of our subjects were healthy young men, it is uncertain whether similar results would be obtained in women, children, or aged men.

To establish clinical relevance, the present pattern of results would have to be verified in vulnerable individuals. In healthy aged people, the diurnal rhythms of water and electrolyte excretion are attenuated such that they experience reduced daytime excretions and increased nighttime excretions relative to young adults^47,48^. The tendency for older people to go to bed earlier than young adults, at a time of day when urine excretory function is still somewhat high, can lead to a need for nighttime voiding, thereby breaking up their sleep. Nocturia, a major problem in aged people, has been found to be associated with an increased risk of mortality^49^. The present results suggest that exposure to ample bright light during the daytime could induce an advance phase shift in the urine excretion rhythm that should alleviate nocturia by enabling more urination before going to bed and thus, perhaps, fewer sleep disruptions. It would be of great interest to determine how daytime light exposure affects the diurnal rhythm of urine excretion in aged people. If it induces a shift like that seen in young men in the present study, then light exposure may represent a new potential remedy for frequent nocturnal urination.

## Supplementary Information

Supplementary Figures.
