# Supplementary material for: Diurnal rhythms of urine volume and electrolyte excretion in healthy young men under differing intensities of daytime light exposure
Source: Sci Rep. 2021 Jun 23;11:13097. doi: 10.1038/s41598-021-92595-0 (PMC8222329; doi:10.1038/s41598-021-92595-0)
Supplement: Supplementary file 1 — Supplementary Figures. [file 41598_2021_92595_MOESM1_ESM.docx]

Supplemental materials

**Diurnal rhythms of urine volume and electrolyte excretion in healthy young men under differing intensities of daytime light exposure**

**Correspondence to:**

Tomoko Wakamura

Human Health Sciences, Graduate School of Medicine, Kyoto University

53 kawahara-cho, shogoin, sakyo-ku, Kyoto, 606-8507, Japan

E-mail: [wakamura.tomoko.5v@kyoto-u.ac.jp](about:blank)

**
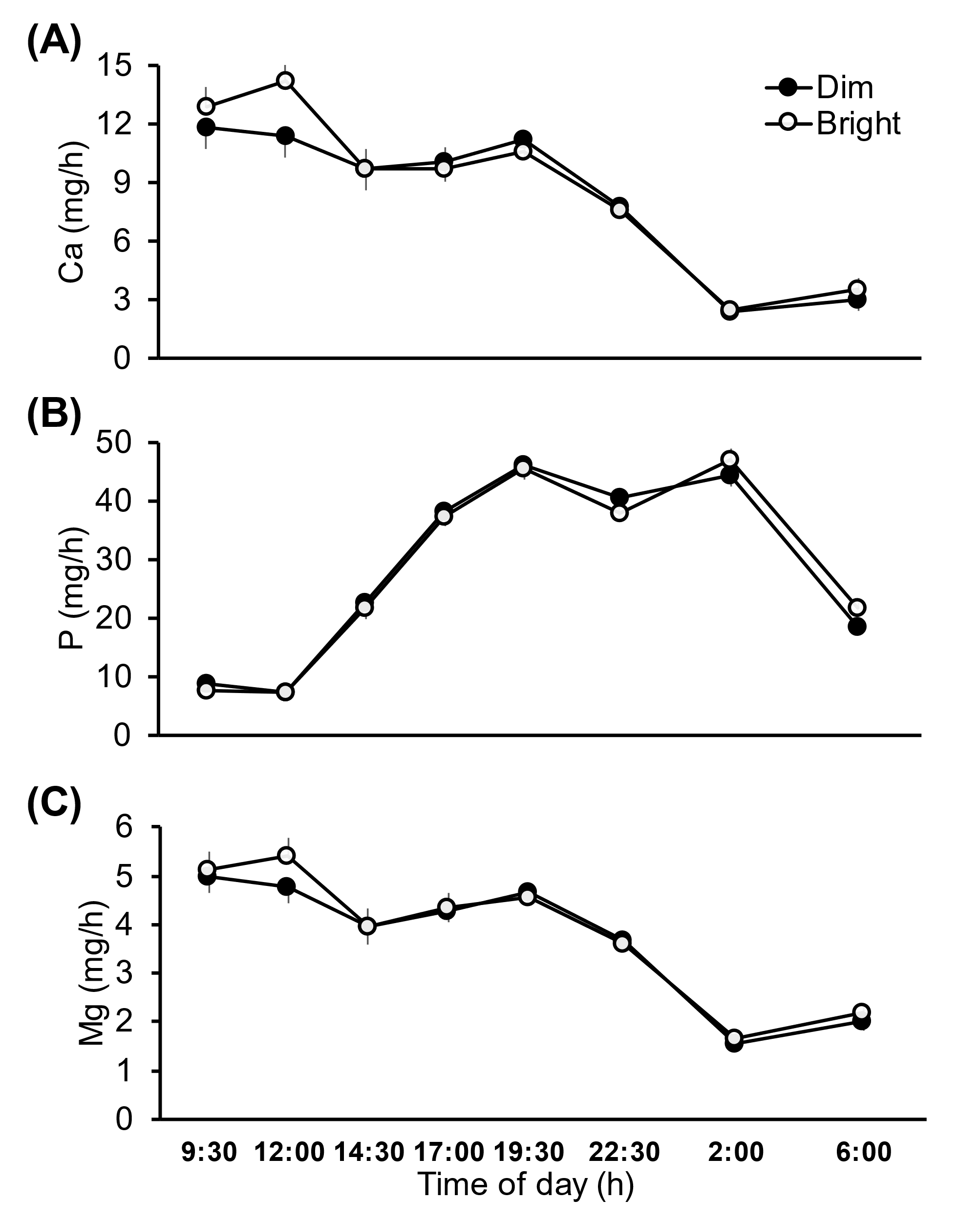
**

**Supplementary Fig. 1.** Effects of bright and dim daytime light exposure on urine excretion variables. **(A)** Ca, **(B)** P, **(C)** Mg. Means ± SEMs are shown. *N* = 19


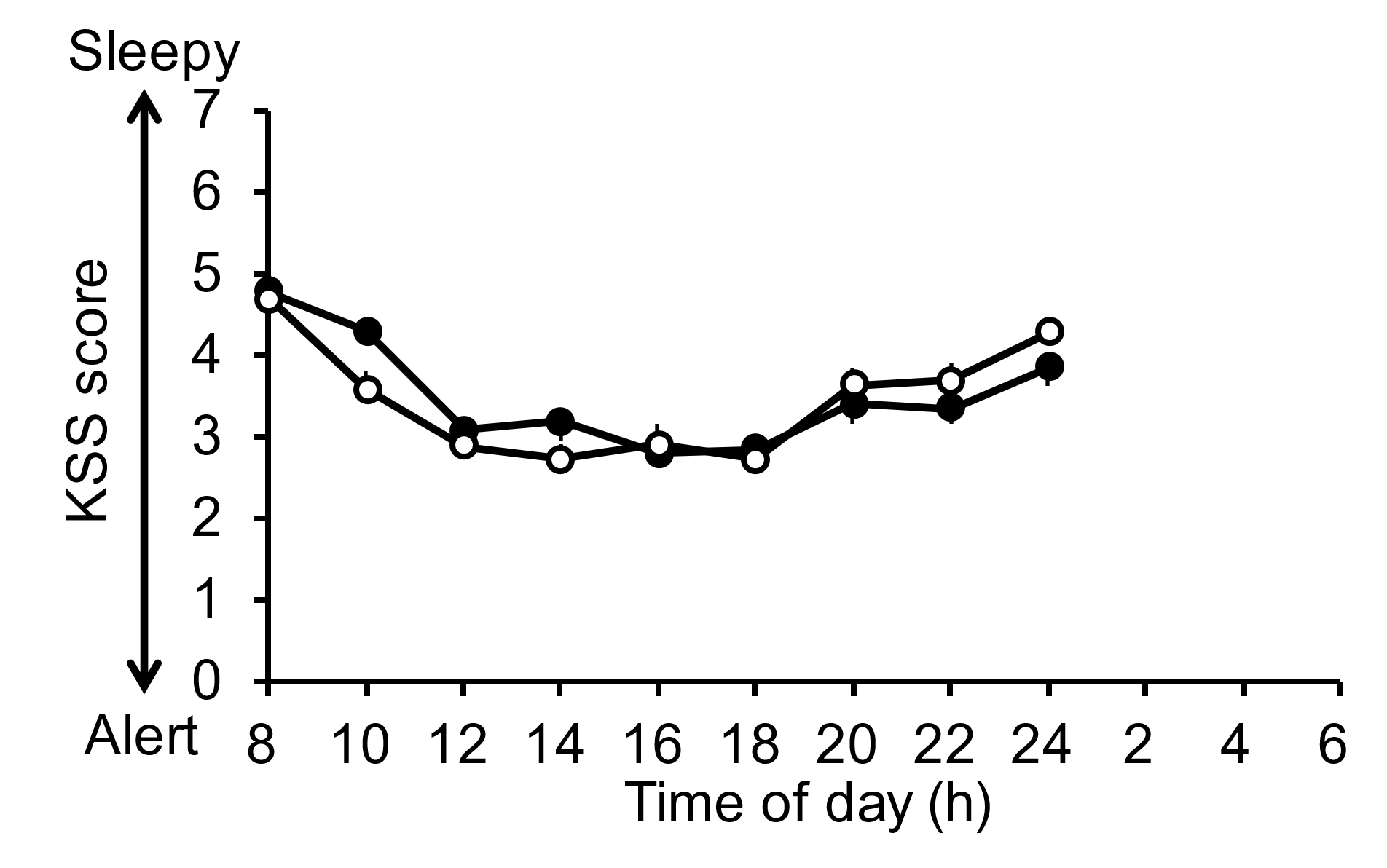
**Supplementary Fig. 2.** Linear mixed model analyses of the effects of time of day and daytime light condition on sleepiness. Sleepiness indexed by KSS scores significantly affected by the time of day, but not by light condition. There was no significant time × light condition interactions; means ± SEMs are shown (*N* = 19).


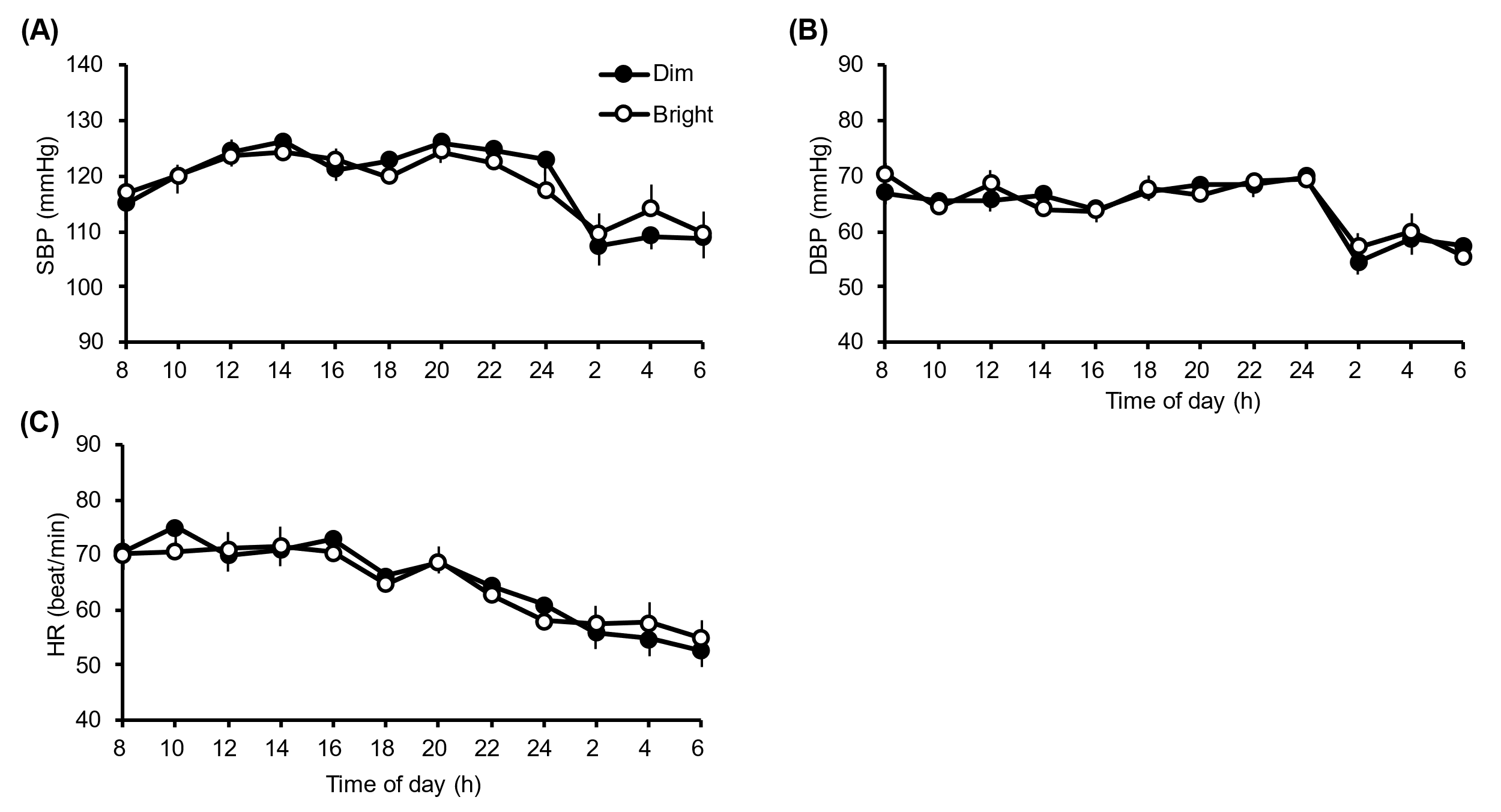
**Supplementary Fig. 3.** Linear mixed model analyses of the effects of time of day and daytime light condition on SBP, DBP, and HR. (A–C) SBP DBP, and HR, respectively, were each very significantly affected by the time of day, but not by light condition. There was no significant time × light condition interactions; means ± SEMs are shown (*N* = 12).
